# Supplementary material for: Tillandsia usneoides Extract Decreases the Primary Tumor in a Murine Breast Cancer Model but Not in Melanoma
Source: Cancers (Basel). 2022 Nov 1;14(21):5383. doi: 10.3390/cancers14215383 (PMC9656114; doi:10.3390/cancers14215383)
Supplement: Supplementary file 1 [file cancers-14-05383-s001.zip › cancers-1958615-supplementary.pdf]

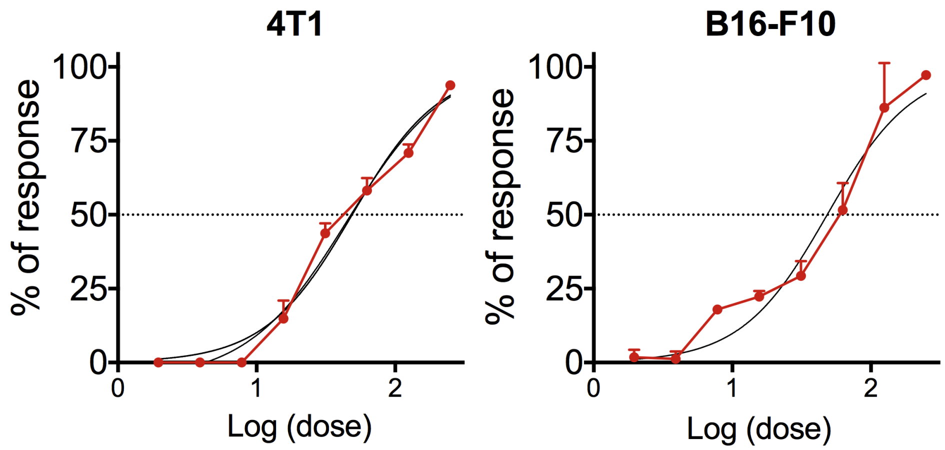

Supplementary Figure S 1. Dose–response viability curve. Cells were seeded in 96-well plates and treated with different concentrations of *T. usneoides* extract (250 a 1.9 µg/mL) for 48 h. Viability were determined by the MTT method described in Materials and Methods. The IC_{50} value was calculated using GraphPad Prism version 8.1.1 for Mac OS X statistics software (GraphPad Software, San Diego, CA). Black line: nonlinear regression curve fitting.

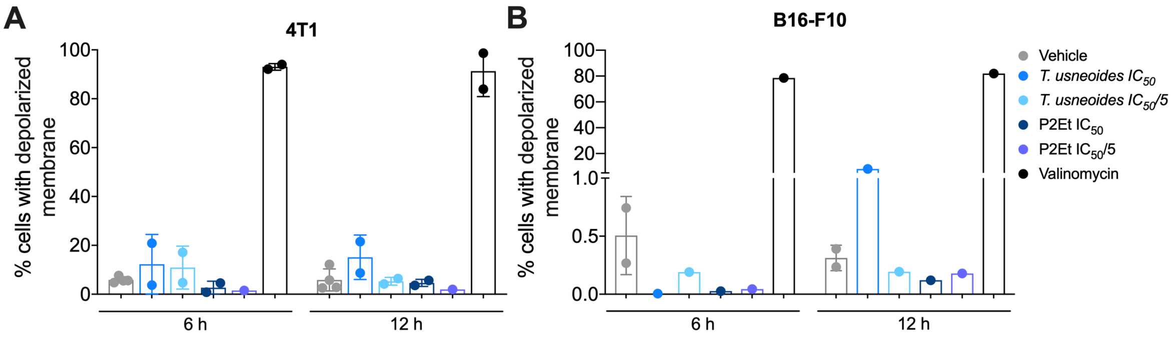

Supplementary Figure S2. *T. usneoides* does not induce mitochondrial depolarization ($\Delta\Psi_m$) **in 4T1 cells and B16-F10 cells. A.** 4T1 cells and **B.** B16-F10 cells. Frequency of cells with depolarized membrane evaluated by flow cytometry after treatment with *T. usneoides* extract IC_{50}, *T. usneoides* extract $IC_{50}/5$, P2Et IC_{50}, P2Et $IC_{50}/5$ or Valynomicin 1 µM (positive control) for 6 and 12h.

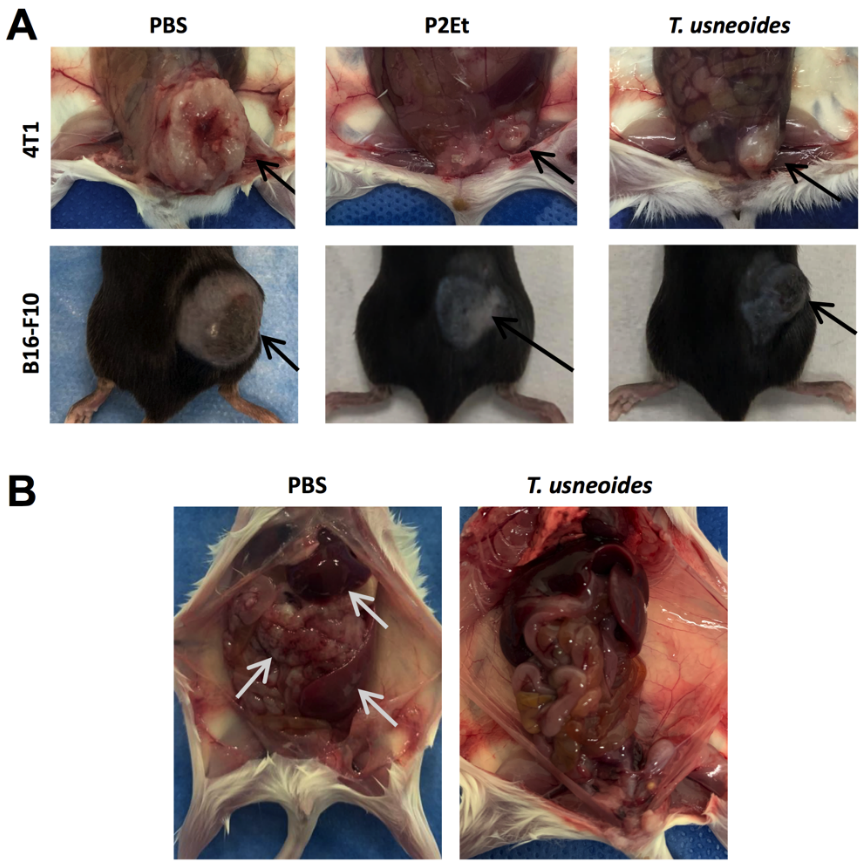

Supplementary Figure S3. Representative images. A. Representative images of tumor size in each treatment group of each tumor model. **B.** Representatives images of reduction of metastasis in BALB/c mice treated with *T. usneoides* extract. Black arrows indicate tumors and white arrows metastases in a PBS-treated mouse.
